# Complete Genome Sequence of Desulfobulbus oligotrophicus Prop6, an Anaerobic *Deltabacterota* Strain That Lacks Mercury Methylation Capability

**DOI:** 10.1128/MRA.00002-21

**Published:** 2021-02-04

**Authors:** Peter T. Podar, Kellie Peyton, Ally Soren, Regina L. Wilpiszeski, Cynthia C. Gilmour, Dwayne A. Elias, Mircea Podar

**Affiliations:** a Biosciences Division, Oak Ridge National Laboratory, Oak Ridge, Tennessee, USA; b School of Engineering, Vanderbilt University, Nashville, Tennessee, USA; c Smithsonian Environmental Research Center, Edgewater, Maryland, USA; University of Southern California

## Abstract

Desulfobulbus oligotrophicus Prop6 is a sulfate-reducing, propionate-oxidizing *Deltabacterota* (formerly *Deltaproteobacteria*) strain from sewage sludge. *Desulfobulbus* species are found in anoxic environments, in animal microbiota, and some produce the neurotoxin methylmercury. The 3.1-Mbp *D. oligotrophicus* genome sequence enables studies of diverse environmental adaptations and the evolutionary genomics of mercury methylation mechanisms.

## Abstract (precis)

Desulfobulbus oligotrophicus strain Prop6 is a sulfate-reducing, propionate-oxidizing deltabacterota from sewage sludge. *Desulfobulbus* species are found in anoxic environments, in animal microbiota, and some produce the neurotoxin methylmercury. The 3.1-Mbp *D. oligotrophicus* genome sequence enables studies of diverse environmental adaptations and the evolutionary genomics of mercury methylation mechanisms.

## ANNOUNCEMENT

*Desulfobulbus* is a genus of sulfate-reducing *Deltabacterota* (formerly Deltaproteobacteria), originally described based on D. propionicus strain lpr3, isolated from freshwater mud (1). Additional species were subsequently characterized from anoxic marine, freshwater, engineered, and human microbiome samples (2–8) and, based on sequence data, are present in many hypoxic environments (9–17). The genome sizes of cultured *Desulfobulbus* strains range from 2.8 Mbp in D. oralis to 5.8 Mbp in D. japonicus, with larger genomes correlating with increased metabolic versatility. Some, but not all, *Desulfobulbus* species encode the enzyme complex HgcAB responsible for the synthesis of methylmercury (18), a powerful neurotoxin that bioaccumulates in trophic chains. The distribution of HgcAB in microbial genomes appears to be driven by both acquisition by horizontal gene transfer and gene loss (19, 20). To better understand the evolution of physiological diversification and its link to mercury methylation, we sequenced the complete genome of D. oligotrophicus isolated from a mesophilic anaerobic sewage sludge digester in Marrakech, Morocco (7).

**FIG 1:**
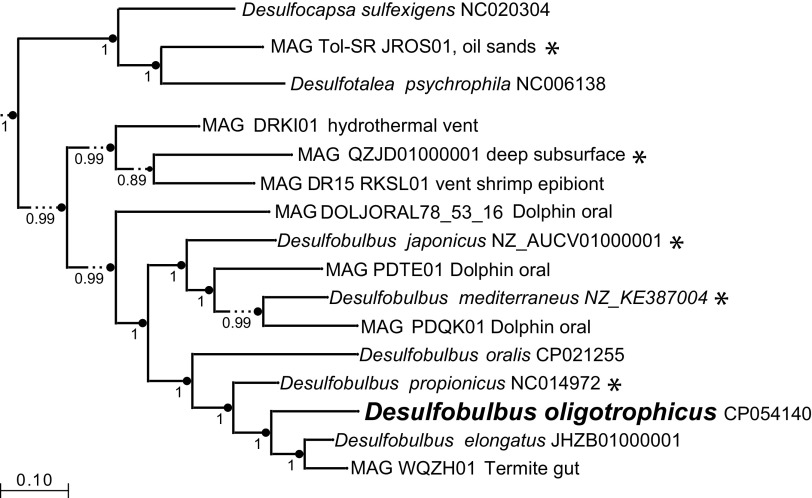
Phylogenetic tree of *D. oligotrophicus* and related bacteria based on 49 core, universal bacterial proteins, using KBase FastTree2. The GenBank accession numbers are listed. Metagenome-assembled genomes (MAGs) and their sources are indicated. Asterisks indicate the presence of the *hgcA* and *hgcB* genes. The numbers at the nodes indicate support values.

*D. oligotrophicus* Prop6^T^ (DSM 103420), obtained from the DSMZ (German Collection of Microorganisms and Cell Cultures, Braunschweig, Germany) was grown for 7 days at 35°C in medium DSM194. Genomic DNA was isolated using proteinase K-SDS digestion, followed by phenol-chloroform extraction as detailed in reference 21, followed by shearing to 10-kb average size using g-TUBEs (Covaris, Woburn, MA). A library prepared with the SMRTbell template prep kit v1.0 (Pacific Biosciences, Menlo Park, CA) was sequenced on a PacBio Sequel instrument. Sequence quality-based filtering and assembly were conducted using the Hierarchical Genome Assembly Process v4 (HGAP4) implemented in the PacBio SMRTLink v7 pipeline, with a target genome size of 4 Mbp. A total of 56,510 polymerase reads (*N*_50_ length, 143,894 nucleotides [nt]) and 478,204 subreads (*N*_50_ length, 10,897 nt) were used in the assembly, resulting in a final polished contig 3,102,012 nt long, with a 1,236-fold mean coverage, a quality value (QV) of 93, and a G+C content of 52.4%. To determine if the chromosome of *D. oligotrophicus* is circular, we designed a pair of oligonucleotides (Doligo forward, 5′-GCGTTTGGGGGTGATGTCTA; Doligo reverse, 5′-CCGCCTCTTATCTTGCCGAT) to amplify outwards from the 5′ and 3′ ends of the contig. Sanger sequencing of the resulting 1.4-kbp PCR product and read mapping using Geneious Prime 2020 (22) identified the correct connection of the contig ends and confirmed that the chromosome is circular. Gene prediction and functional annotation were generated with NCBI’s Prokaryotic Genome Annotation Pipeline (PGAP) v4.8 (23), which identified 2,721 protein coding sequences, 48 tRNAs, 2 rRNA operons, and 4 noncoding RNAs (ncRNAs). The genome sequence lacks the *hgcA* and *hgcB* genes, and experimental analysis using an established protocol (24) confirmed that *D. oligotrophicus* does not methylate inorganic mercury. Based on a genomic tree constructed in KBase (25) under SpeciesTreeBuilder v1.0, *D. oligotrophicus* is most closely related to *D. propionicus* and *D. elongatus* (Fig. 1).

### Data availability.

The Desulfobulbus oligotrophicus Prop6^T^ (DSM 103420) genome sequence has been deposited in GenBank under the accession number CP054140. The version described in this paper is the first version, CP054140.1. The PacBio reads have been deposited in the SRA under the accession number SRX9754829.
